# Genotoxic responses to titanium dioxide nanoparticles and fullerene in *gpt *delta transgenic MEF cells

**DOI:** 10.1186/1743-8977-6-3

**Published:** 2009-01-20

**Authors:** An Xu, Yunfei Chai, Takehiko Nohmi, Tom K Hei

**Affiliations:** 1 Center for Radiological Research, College of Physicians & Surgeons, Columbia University, New York, New York, USA; 2 Key Laboratory of Ion Beam Bioengineering, Institute of Plasma Physics, Chinese Academy of Sciences, Hefei, Anhui, PR China; 3 Department of Environmental Health Sciences, Mailman School of Public Health, Columbia University, New York, New York, USA; 4 Division of Genetics and Mutagenesis, National Institute of Health Science, Tokyo, Japan

## Abstract

**Background:**

Titanium dioxide (TiO_2_) nanoparticles and fullerene (C_60_) are two attractive manufactured nanoparticles with great promise in industrial and medical applications. However, little is known about the genotoxic response of TiO_2 _nanoparticles and C_60 _in mammalian cells. In the present study, we determined the mutation fractions induced by either TiO_2 _nanoparticles or C_60 _in *gpt *delta transgenic mouse primary embryo fibroblasts (MEF) and identified peroxynitrite anions (ONOO^-^) as an essential mediator involved in such process.

**Results:**

Both TiO_2 _nanoparticles and C_60 _dramatically increased the mutation yield, which could be abrogated by concurrent treatment with the endocytosis inhibitor, Nystatin. Under confocal scanning microscopy together with the radical probe dihydrorhodamine 123 (DHR 123), we found that there was a dose-dependent formation of ONOO^-^ in live MEF cells exposed to either TiO_2 _nanoparticles or C_60_, and the protective effects of antioxidants were demonstrated by the nitric oxide synthase (NOS) inhibitor, N^G^-methyl-L-arginine (L-NMMA). Furthermore, suppression of cyclooxygenase-2 (COX-2) activity by using the chemical inhibitor NS-398 significantly reduced mutation frequency of both TiO_2 _nanoparticles and C_60_.

**Conclusion:**

Our results provided novel information that both TiO_2 _nanoparticles and C_60 _were taken up by cells and induced kilo-base pair deletion mutations in a transgenic mouse mutation system. The induction of ONOO^- ^may be a critical signaling event for nanoparticle genotoxicity.

## Background

Nanoparticles are referred to a class of particles with properties distinctively different from their bulk and molecular counterparts [1,2]. Due to the unique electrical, thermal, mechanical, and imaging properties, manufactured nanoparticles are highly desirable to improve the quality and performance of materials in a diverse array of industrial and medical applications, ranging from biomedicine, nanoelectronics and mechanical engineering [3,4]. However, with the increase in large scale production of manufactured nanoparticles, the potential occupational and public exposure to manufactured nanoparticles has aroused concern because of their large surface areas and the ability to deposit in the body [5]. Thus, a comprehensive study is clearly needed to fully explore the genotoxicity of manufactured nanoparticles, which may help to better understand their deleterious health effects and create environmentally friendly and biologically relevant nanoparticles.

Among the manufactured nanoparticles, titanium dioxide (TiO_2_) nanoparticles have been already in mass production for decades. In the early years, TiO_2 _with the usual size of > 100 nm is considered a poorly soluble particulate and has been widely used as an additive in the production of a white pigment, food colorant, sunscreens, and cosmetic creams by virtue of its biologically inert mess in both humans and animals [6-8]. Recent evidence, however, has suggested that nano-sized TiO_2 _can cause inflammatory response in airways of rats and mice, fibrosis or lung tumors in rats, and DNA damage in Chinese hamster ovary (CHO) cells, Syrian hamster embryo fibroblasts and human lymphoblastoid cells [9-12]. A significant decrease in the level of glutathione was observed in rat lung alveolar macrophage following exposure to TiO_2 _nanoparticles, indicating the induction of reactive oxygen species (ROS) [13]. Furthermore, exposure of human bronchial epithelial cells to TiO_2 _nanoparticles was shown to induce oxidative DNA damage, micronuclei formation, and increases in the levels of hydrogen peroxide (H_2_O_2_) and nitric oxide (NO) [14]. Although various *in vivo *and *in vitro *studies have shown that TiO_2 _nanoparticles are more toxic than its larger, micron-size counterparts, the molecular mechanisms responsible for the genotoxicity in nano-sized TiO_2 _are not yet understood.

Compared to TiO_2 _nanoparticles that have been used for over half a century, fullerene (C_60_) is a novel carbon allotrope, which was discovered in 1985 and consist of a polygonal structure made up solely with 60 carbon atoms. In the past few years, methods was established to considerably improve its mass production capacity [15]. Currently, C_60 _with spherical symmetry has aroused intense interest for its multi-functional uses in materials science and optics and is considered for a variety of biological applications, such as imaging probes and drug carriers. Although investigation of the biological properties of pure, underivatized C_60 _has been hampered by its low aqueous solubility, C_60 _is lipophilic and can be localized in the lipid-rich regions including cell membrane *in vitro *[16]. It has been reported that equivalent doses of an aggregated form of underivatized C_60 _are 3–4 orders of magnitude more toxic to human dermal fibroblasts, lung epithelial cells, and normal human astrocytes than the derivatized, highly water-soluble derivative, C_60_(OH)_24_. The increased toxicity is thought to be mediated through ROS induced lipid peroxidation of cell membrane [17]. In accordance with these data, a study performed using the largemouth bass reveals significant lipid peroxidations in brains of this aquatic species after exposure to underivatized C_60 _[18]. In a recent study, Isakovic et al. confirmed the greater toxicity of C_60 _in a variety of cell lines [19]. Nevertheless, there is considerable evidence that C_60 _induces slightly toxic in bacteria, rats, as well as in murine and human macrophages [20,21]. Thus, to define and constrain the potential biomedical applications of C_60_, it is of great interest to identify the genotoxicity of C_60 _in mammalian cells.

In the present study, we assessed the genotoxicity of TiO_2 _particles of different size distributions and C_60 _using *gpt *delta transgenic mouse primary embryo fibroblasts (MEF) [22-24]. We investigated the mutation frequencies at both the *redBA *and *gam *loci and the contribution of endocytosis to the mutagenic process. Since oxidative stress has been widely implicated as a probable mechanism of genotoxicity for a variety of environmental mutagens that induce reactive oxygen and nitrogen species (ROS/RNS) under either endogenous or exogenous insults [25,26], the contributions of peroxynitrite anions (ONOO^-^) and cyclooxygenase-2 (COX-2) were determined in the genotoxic response to TiO_2 _nanoparticles and C_60_. Our results provided direct evidence that both TiO_2 _nanoparticles and C_60 _induced kilobase pair deletion mutations in mammalian cells that were mediated by ONOO^.^. Furthermore, COX-2 signaling pathway, which is essential in mediating cellular inflammation, carcinogenesis, and genomic instability, might be a critical signaling event for nanoparticle genotoxicity.

## Methods

### MEF cell culture

*gpt *delta transgenic mice were mated, and pregnant females were sacrificed on day 14 of the gestation period. The use of the transgenic animals and the experimental protocol were previously approved by the Columbia University Institutional Animal Care and Use Committee. The animals were treated humanely and with regard towards the alleviation of pain and suffering. The embryos were surgically removed and embryonic tissue prepared in culture according to standard procedures [27]. These cultures were grown and maintained in Dulbecco's modified Eagle's medium (Gibco-BRL) containing 15% heat-inactivated fetal bovine serum and penicillin (100 U/ml), streptomycin (50 μg/ml) in a 5% CO_2 _environment at 37°C.

### Preparation of aqueous dispersion of TiO_2 _nanoparticles and C_60_

Anatase TiO_2 _particles with different sizes were used in the present study. TiO_2 _nanoparticles with an average primary particle diameter of either 5 nm (99.7% purity, referred to as TiO_2 _5 nm) or 40 nm (99.9% purity, referred to as TiO_2 _40 nm) were purchased from Sigma-Aldrich (St. Louis, MO, USA) and Inframat Advanced Materials LLC (Farmington, CT, USA), respectively. We purchased the commercially available TiO_2 _at -325 mesh in diameter (≥ 99% purity, referred to as TiO_2 _-325 mesh) from Sigma-Aldrich (St. Louis, MO, USA). Pure (99.5%) C_60 _(referred as to C_60_) was obtained from SES Research (Houston, TX, USA). The BET Surface Area for 5 nm, 40 nm, and 325 mesh TiO_2 _was 114.1261 m^2^/g. 38.2268 m^2^/g, and 8.9146 m^2^/g, respectively, which was determined by ASAP 2020 Accelerated Surface Area and Porosimetry (Micromeritics, Norcross, GA 30093, USA). The above materials were used as received, and no further modifications were applied. TiO_2 _particles were suspended in distilled water to a desired concentration and sterilized by heating to 120°C for 30 min. C_60 _suspension was prepared by long-term (60 days) stirring in water and sterilized by autoclaving. Before being diluted with 5 ml tissue culture medium for cell treatment in T-25 flasks, all particles were sonicated on ice for 30 min to ensure a uniform suspension. For all experiments and analysis, distilled water was filtered with a 0.45 mm nominal pore size polycarbonate syringe filter (Millipore, MA, USA).

### Treatment with inhibitors

Nystatin (Sigma-Aldrich, St. Louis, MO, USA), an endocytosis inhibitor, was diluted directly from stock solution with medium to a final concentration of 10 U/ml. N^G^-methyl-L-arginine (L-NMMA; Molecular Probes, Inc., Eugene, OR, USA), nitric oxide synthase inhibitor, was dissolved in distilled water (10 mM stock) and filter sterilized. Stock L-NMMA was diluted with medium to a final concentration of 500 μM and added to the cultures 24 h before particle treatment and remained in the medium or buffer throughout the treatment period. NS-398 (Cayman Chemical, Ann Arbor, MI, USA), a selective inhibitor of cyclooxygenase-2 (COX-2), was dissolved in dimethyl formamide to a desired stock concentration. Stock NS-398 solution was diluted with medium to a working concentration of 50 μM.

### Cytotoxicity assay

Cell viability was evaluated by MTT assay based on the ability of viable cells to convert a water-soluble tetrazolium salt into a water-insoluble formazan product [28]. The enzymatic reduction of the tetrazolium salt happens only in living, metabolically active cells but not in dead cells. Cultures were incubated in two-well chamber slides at a density of 5.0 × 10^5 ^cells per well at 37°C for 24 h. Graded doses of particles were added to the culture medium and incubated for another 24 h. At the end of the treatment period, the medium was removed and 200 μl of 5 mg/ml MTT was added into each well and the cultures were incubated for another 4 h. The supernatant was removed and 1 ml acidic isopropanol was added to dissolve the formazan crystals. The absorbance at 570 nm was determined by a spectrophotometer.

### Genomic DNA isolation

Genomic DNA was isolated from MEF cells using the RecoverEase™ DNA isolation kit (Stratagene, La Jolla, CA, USA) according to the protocol developed by the supplier. Briefly, about 5.0 × 10^6 ^cells were transferred to a chilled Wheaton dounce tissue grinder and the homogenate obtained was filtered and centrifuged at 1100 × g for 12 min at 4°C. The pellet was resuspended in digestion buffer containing RNAses (RANse-It™, Stratagene) containing proteinase K solution (2 mg/ml pre-warmed to 50°C). Using wide-bore pipette tips, the samples were transferred to dialysis cups floating on the surface of TE buffer (500 ml) and dialyzed for 24 h. The purity and concentration of DNA was checked spectrophotometrically and samples were diluted with TE buffer to a final DNA concentration of about 0.5 mg/ml, and stored at 4°C for up to 3 months prior to mutation analysis.

### *In vitro *packaging of DNA

The λ-DNA was recovered from approximately 5 μg of genomic DNA and packaged with terminase and phage proteins contained in the Transpack™ kit (Stratagene, La Jolla, CA, USA) to produce infectious λ-phages. Viable phages were infected into *E. coli *XL-1 Blue MRA (Stratagene, La Jolla, CA, USA), mixed with lambda-trypticase agarose and poured onto 100 mm plates containing 30 ml bottom agar. Plates were incubated overnight at 37°C. The average of rescued phages per packaging reaction was 1.8 × 10^6 ^in the present studies. There was no significant difference in the titers between control and exposed groups.

### Spi^- ^mutation analysis

The mutant frequencies at *red/gam *loci were determined by Spi^- ^selection as described previously [24,29,30]. Briefly, packaged phages were infected into *E. coli *XL-1 Blue MRA (P2) (Stratagene, La Jolla, CA, USA). Infected cells were mixed with molten soft agar, poured onto lambda-trypticase agar plates and incubated at 37°C. The plaques detected on the plates (Spi^- ^candidates) were suspended in 50 μl of SM buffer. The suspension was spotted on the two types of plates where *E. coli *XL-1 Blue MRA (P2) or WL95 (P2) strain was spread. The plates were incubated for 24 h at 37°C. The numbers of mutants that made clear spots on both strains were counted as confirmed Spi^- ^mutants. Mutation frequencies were calculated by comparing the titration and number of confirmed mutant plaques.

### Quantification of cell-particle interaction

Based on the principle of flow cytometry technology, the sizes and the shapes of all the cells can be determined by the measurement of forward scattered (FSC) and side scattered (SSC) lights [31]. Generally, FSC is related to the cell size and the optical refraction index of the outer membrane of the cells, whereas SSC indicates surface or cellular granularity. Exponentially growing MEF cells were exposed to graded doses of particles for 24 h. After treatment, cells were rinsed with balanced salt solution and fixed. The uptake of particles were determined by flow cytometry (Becton Dickinson, San Jose, CA) equipped with an air-cooled laser providing 15 mW at 488 nm.

### Measurement of peroxynitrite anions (ONOO^-^) in particles treated cells

DHR123 is a nonfluorescent, noncharged dye that easily penetrates cell membrane. Once inside the cell, DHR123 selectively reacts with peroxynitrite to yield rhodamine 123, a highly fluorescent compound, which subsequently accumulates in the mitochondria [32]. Exponentially growing MEF cells (2 × 10^5 ^cells) grown on 35 mm glass bottom microwell dishes (DTC3 dishes, BiopTechs) were pretreated for 30 min with a 5 μM dose of dihydrorhodamine 123 in ACAS buffer (127 mM NaCl, 0.8 mM KCl, 1.2 mM CaCl_2_, 1.2 mM KH_2_PO_4_, 4.4 mM C_6_H_12_O_6_, 10 mM HEPES, pH 7.4) at 37°C. Graded doses of particles, with or without L-NMMA, were then added to the cultures. The fluorescence of rhodamine 123 in cultures was measured using a confocal microscope and a semi-quantitative estimation of the fluorescent signal was obtained using the composite images generated by Adobe Photoshop (Adobe Systems, Inc., San Jose, CA) as described above.

### Statistical analysis

All numerical data were calculated as mean and standard deviation (S.D.) and evaluated by Student's *t-test*. Difference between groups was considered significant when p < 0.05.

## Results

### TiO_2 _particles and C_60 _induced cytotoxicity in transgenic MEF cells

**Figure 1:**
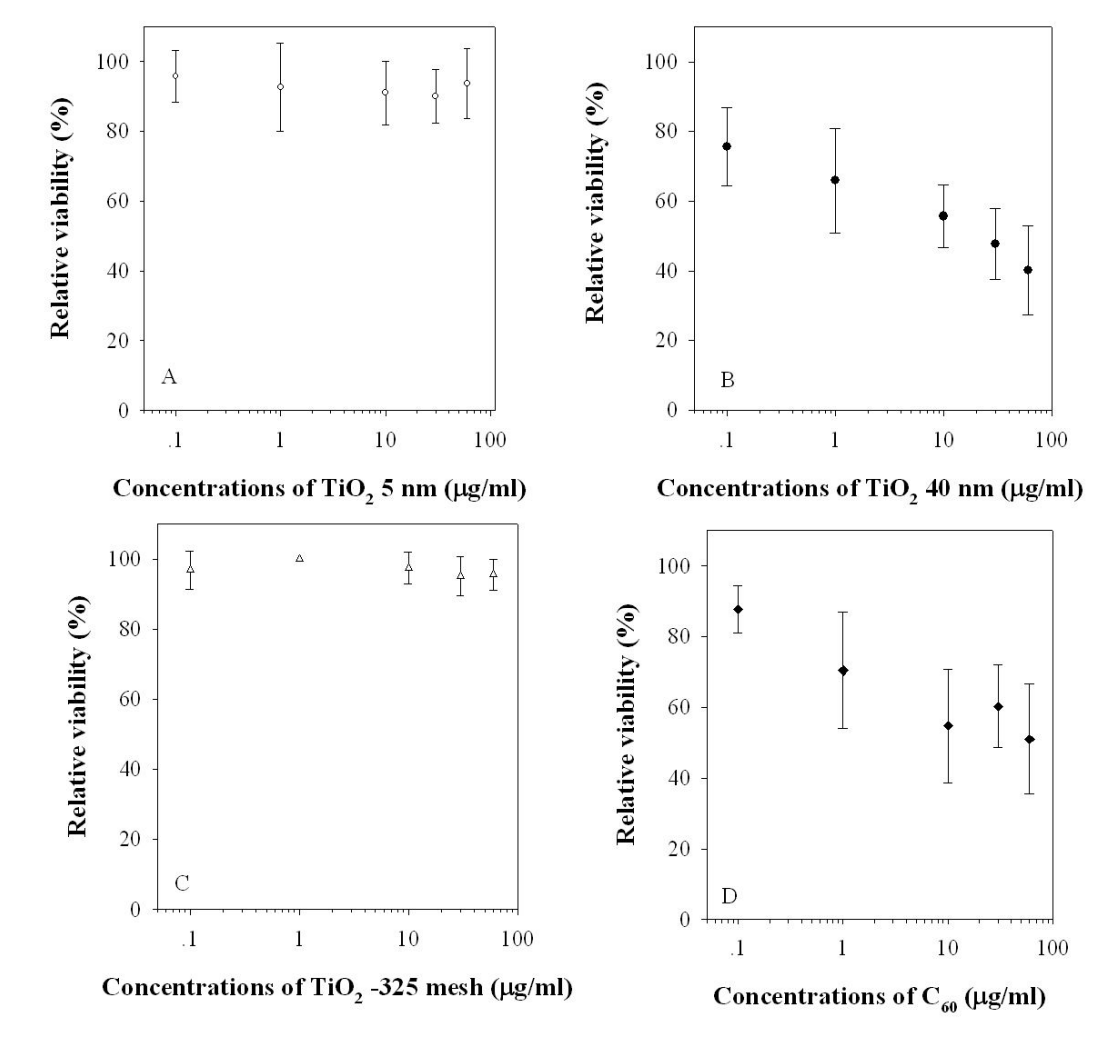
**Cell viability of transgenic MEF cells treated with graded doses of TiO_2 _particles and C_60 _for 24 h**. Data were the average of three independent experiments. Bar: ± SD.

The viability of MEF cells exposed to graded doses of either TiO_2 _particles or C_60 _was analyzed by using the MTT assay. As shown in Figure 1A–C, exposure of MEF cells to different particle sizes of TiO_2 _at doses ranging from 0.1 to 30 μg/mlfor 24 h produced various dose response curves in cell viability. Addition of either TiO_2 _5 nm or TiO_2 _-325 mesh to the culture medium had essentially no effect on the viability of MEF cells. In contrast, treatment of MEF cells with TiO_2 _40 nm resulted in a dose-dependent decrease in cell viability. The viability of MEF cells was reduced by 24%, 34%, 44%, 52%, and 60%, when the concentrations of TiO_2 _particles were 0.1, 1, 10, 30 and 60 μg/ml, respectively. The LD_50 _dose of TiO_2 _40 nm, which resulted in 50% cell killing, was about 30 μg/ml. Likewise, there was a dose-dependent decrease of cell viability of MEF cells treated with C_60 _at doses ranging from 0.1 μg/ml to 10 μg/ml (Figure 1D). However, there was no further decrease in cell viability with C_60_ concentration > 10 μg/ml.

### Mutation frequencies at *red/gam *gene loci were determined in response to either TiO_2 _particles or C_60 _exposure

**Figure 2:**
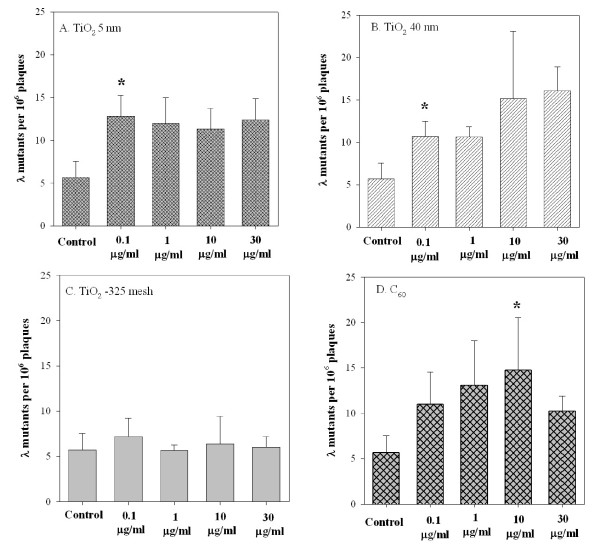
**Mutagenic potential of different particle sizes of TiO_2 _and C_60_ at *redBA *and *gam *loci in transgenic MEF cells**. 5 × 10^5 ^MEF cells were treated with graded doses of either different particle sizes of TiO_2 _or C_60 _as described in the text. Results were expressed as the total number of confirmed λ mutants divided by the total number of rescued phages. The average number of preexisting mutants per 10^6 ^plaques used for these experiments was 5.69 ± 1.87. Data were pooled from 3 independent experiments. Bars, ± SD. * indicated p < 0.05.

To investigate the mutagenicity of TiO_2 _and C_60 _in the *gpt *delta assay, a Spi^- ^mutation assay was used to determine the mutation frequencies induced by either TiO_2 _or C_60 _exposure for 3 days in transgenic MEF cells. The average number of spontaneous *red/gam *gene mutants per 10^6 ^recovered plaques in MEF cells used for these experiments was 5.69 ± 1.87. In cells treated with a dose of 0.1 μg/ml TiO_2 _5 nm, there was a 2.2-fold increase in mutation yield above the background (Figure 2A). However, with further increase in the concentration of TiO_2 _5 nm, there was no further increase in mutant yield. In contrast, treatment of MEF cells with TiO_2 _40 nm resulted in a dose-dependent induction of mutation yield atthe *red/gam *gene locus (Figure 2B). A significant increase in mutation yield over the background level was observed at TiO_2 _40 nm at concentrations ≥ 0.1 μg/ml (p < 0.05). The mutant fraction in cells treated with a dose of 10 μg/ml of TiO_2 _40 nm was 2.7-fold higher than background. In contrast, the mutation yield at the *red/gam *gene locus was not much altered by TiO_2 _-325 mesh at doses ranging from 0.1 μg/ml to 30 μg/ml (Figure 2C). A clear dose-dependent induction of mutation at the *red/gam *gene locus was observed when MEF cells were subject to C_60 _treatment at doses ranging from 0.1 μg/ml to 30 μg/ml (Figure 2D). There was a 2.6-fold increase in the mutation yield in cells treated with C_60 _at a concentration of 10 μg/ml (p < 0.05). These results indicated that TiO_2 _nanoparticles and C_60 _were able to produce deletion mutations in *gpt *delta transgenic mutation assay system.

### Quantification of TiO_2 _particles and C_60 _uptake

**Figure 3:**
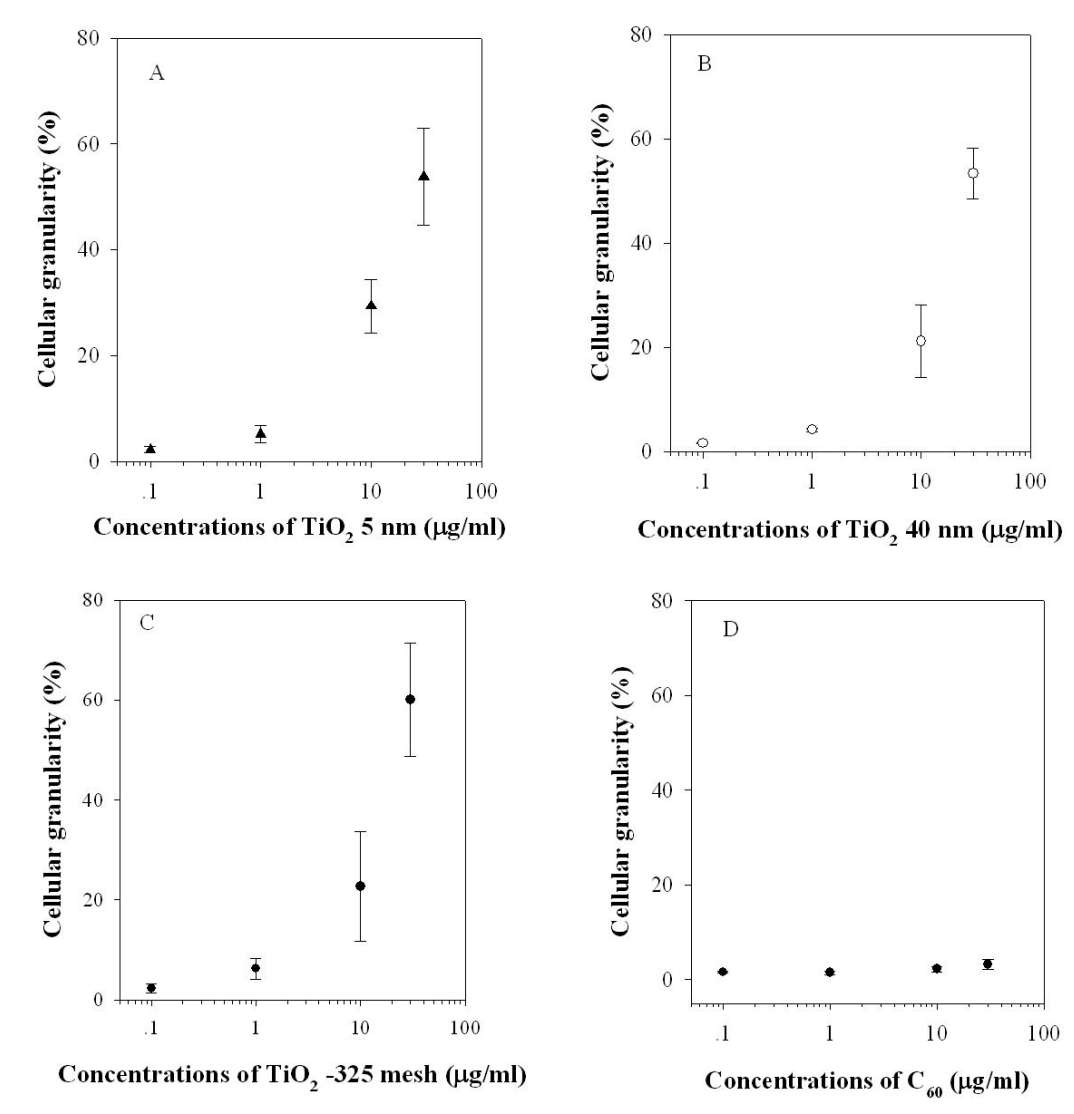
**Effect of TiO_2 _and C_60 _on the cellular granularity**. MEF cells were exposed to graded doses of either different particle sizes of TiO_2 _or C_60 _for 24 h. After treatment, cells were detected and quantified by flow cytometry. Data were pooled from 3 independent experiments. Error bars indicated S.D.

The elastically scattered light from cells/tissues provides a convenient and non-invasive approach to monitor morphological parameters and structural modifications of cells/tissues. The relative intensity of forward scattered (FSC) and the side scattered (SSC) light from single cell is often used in flow cytometry for qualitative measurement of size and granularity of cells. There were significant increases in cellular granularity induced by different particle size of TiO_2 _in an exponentially, dose-dependent manner (Figure 3 A–C). However, it was difficult to quantify C_60 _uptake. These results were consistent with the findings that TiO_2 _particles were taken into phagosomes while C_60 _was difficult to visualize under the electronic microscopy [33].

### Effect of endocytosis inhibitor on TiO_2 _particles and C_60 _induced genotoxicity in MEF Cells

**Figure 4:**
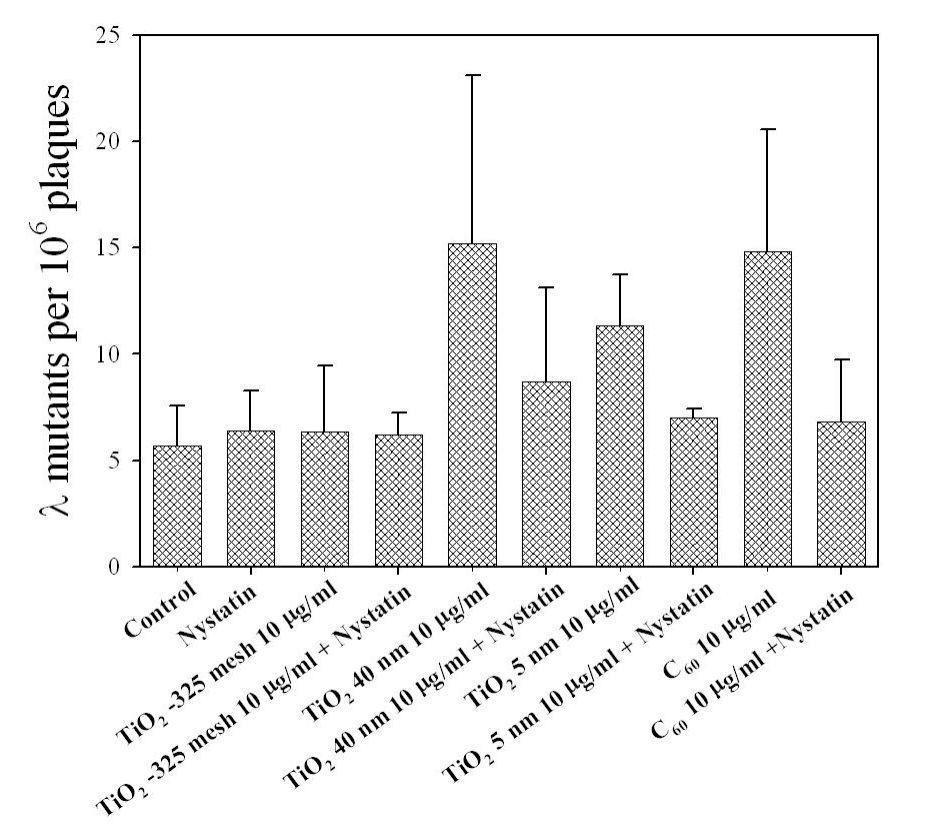
**Mutation fractions at *redBA/gam *loci in MEF cells exposed to either TiO_2 _particles or C_60 _at a dose of 10 μg/ml either in the presence or absence of Nystatin (10 U/ml), an endocytosis inhibitor**. Results were expressed as the total number of confirmed λ mutants divided by the total number of rescued phages. The average number of preexisting mutants per 10^6 ^plaques used for these experiments was 5.69 ± 1.87. Data were pooled from 3 independent experiments. Bars, ± SD.

To determine the particle uptake effect on the genotoxicity of either TiO_2 _particles or C_60_, Nystatin, an endocytosis inhibitor which disrupts internalization via caveolae, was used in the present experiments [34]. As shown in Figure 4, the Spi^- ^mutant yields in MEF cells induced by either TiO_2 _5 nm, TiO_2 _40 nm or C_60 _at a concentration of 10 μg/ml were suppressed in the presence of 10 U/ml Nystatin by 1.6-fold, 1.8-fold and 2.2-fold, respectively. However, the presence of Nystatin had no effect on the mutation yield induced by TiO_2 _-325 mesh treatment. The dose of Nystatin used in these experiments was non-cytotoxic nor mutagenic.

### TiO_2 _particles and C_60 _stimulated peroxynitrite anion (ONOO^-^) production in MEF cells

**Figure 5:**
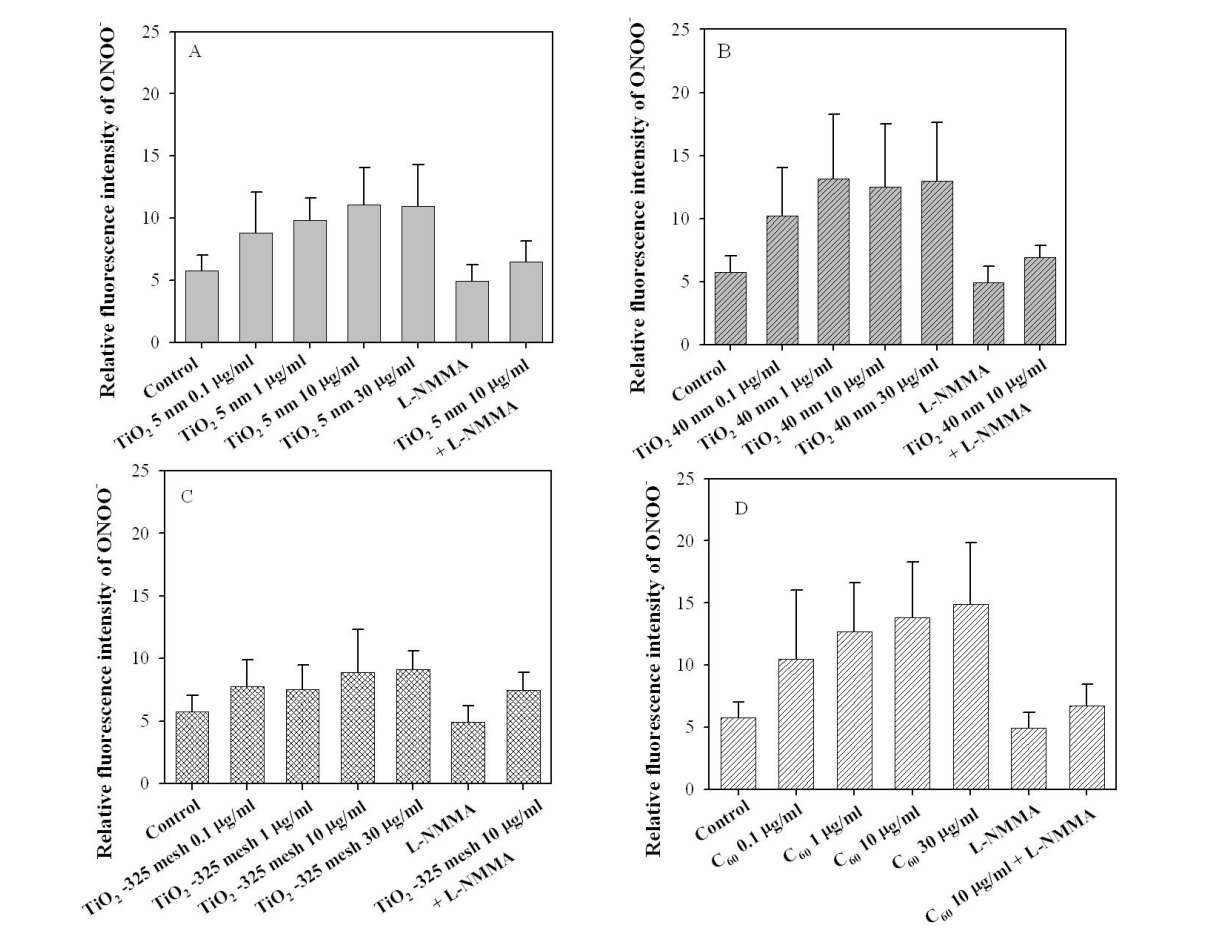
**Generation of ONOO^-. ^in MEF cells treated with graded doses of either TiO_2 _particles or C_60 _in the presence or absence of L-NMMA at a dose of 500 μM**. The fluorescence signals generated from composite images were obtained by confocal microscopy in MEF cells preloaded with dihydrorhodamine 123 with or without subsequent either TiO_2 _particles or C_60 _treatment. The relative fluorescence intensity of ONOO^-. ^in MEF cells as a function of either TiO_2 _particles or C_60 _concentration with or without L-NMMA. The relative intensities are expressed in arbitrary units. Data were pooled from three independent experiments. Bars, ± S.D.

ONOO^- ^is a strong oxidant and nitrating species resulting from the near diffusion-controlled reaction of superoxide with NO. Treatment of MEF cells with either TiO_2 _5 nm, TiO_2 _40 nm, or C_60 _resulted in a dose-dependent induction of ONOO^- ^(Figure 5A, B, and 5D). The fluorescent intensity in cells treated with a 10 μg/ml dose of TiO_2 _5 nm was 1.9-fold higher than the background (p < 0.05) (Figure 5A). A significant increase in fluorescent intensity over the background level was observed with either TiO_2 _40 nm or C_60 _at concentrations > 1 μg/ml (p < 0.05) (Figure 5B, D). For example, the average fluorescent intensity in cells treated with either TiO_2 _40 nm or C_60 _at a dose of 10 μg/ml was 2.2-fold and 2.4-fold above nontreated cells, respectively. It should be noted that the fluorescent intensity obtained in cells treated with TiO_2 _-325 mesh was slightly higher than the background level; however, the difference was not statistically significant (Figure 5C). In the presence of N^G^-methyl-L-arginine (L-NMMA), which has been shown to competitively block the activity of NOS in various cell lines, the fluorescent signals in either TiO_2 _nanoparticle-treated or C_60_-treated cells were suppressed significantly (p < 0.05).

### Effects of NOS inhibitor on TiO_2 _particles and C_60_-induced genotoxicity in MEF cells

**Figure 6:**
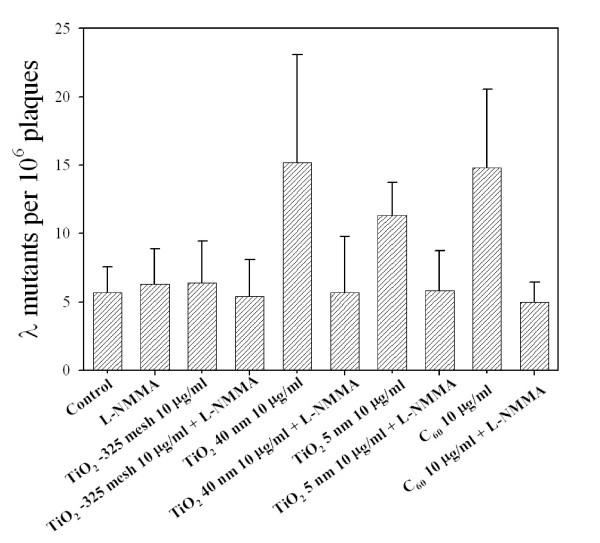
**Mutant fractions at *redBA/gam *loci in MEF cells exposed to either TiO_2 _particles or C_60 _at a dose of 10 μg/ml in the presence or absence of L-NMMA (500 μM)**. Data were pooled from three independent experiments. Error bars indicate ± SD.

The nitric oxide synthases (NOS) are hemoproteins with a cytochrome P450-like active site that catalyze the oxidation of arginine to nitric oxide and citrulline [35]. To evaluate the contribution of ONOO^- ^in TiO_2 _and C_60 _mutagenesis, MEF cells were exposed to either TiO_2 _particles or C_60 _either in the presence or absence of L-NMMA (Figure 6). Concurrent treatment of MEF cells with either TiO_2 _40 nm, TiO_2 _5 nm or C_60 _at a dose of 10 μg/ml and L-NMMA at a concentration of 500 μM dramatically suppressed the mutation yield by 2.7-fold *(column 5 versus 6)*, 1.9-fold *(column 7 versus 8)*, and 3-fold *(column 9 versus 10)*, respectively (p < 0.05). Consistent with our previous studies, treatment of MEF cells with TiO_2 _-325 mesh resulted in little or no Spi^- ^mutations. Addition of L-NMMA (500 μM) had no effect on the overall mutation yield induced by TiO_2 _-325 mesh *(column 3 versus 4)*. The dose of L-NMMA used here has been shown to be non-toxic and non-mutagenic in mammalian cells. These results strongly suggested that RNS, and especially ONOO^-^, were causally linked to the mutagenic response of both TiO_2 _nanoparticle and C_60 _exposure.

### Effects of COX-2 inhibitor on TiO_2 _particle and C_60_-induced genotoxicity in MEF cells

**Figure 7:**
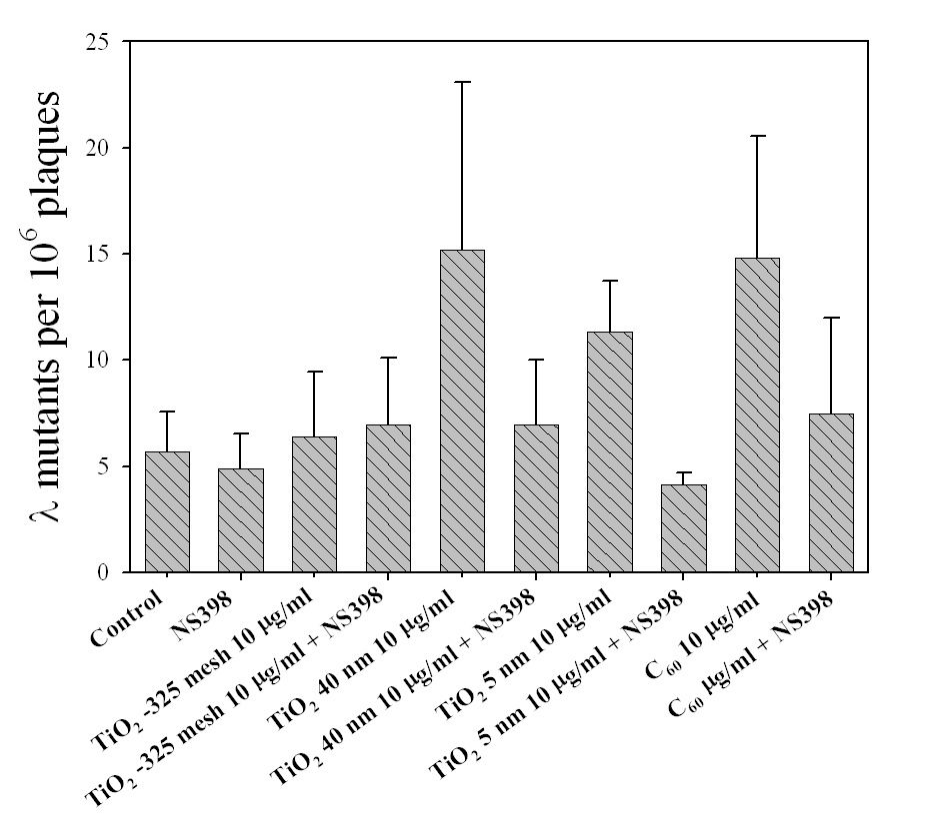
**Mutant fractions at *redBA/gam *loci in MEF cells exposed to either TiO_2 _particles or C_60 _at a dose of 10 μg/ml in the presence or absence of NS398 (50 μM)**. Data were pooled from three independent experiments. Error bars indicate ± SD.

Nitric oxide synthase, which is critical to the biosynthesis of ONOO^-^, has been shown to be involved in the regulation of COX-2 expression [36]. Figure 7 showed the effect of a noncytotoxic and nonmutagenic dose of NS398, a specific inhibitor of COX-2 activity, on either TiO_2 _particles or C_60_ mutagenesis at *redBA/gam *loci in MEF cells. Treatment of cells with a 10 μg/ml dose of either TiO_2 _40 nm, TiO_2 _5 nm, or C_60 _resulted in mutant fractions of 15.2 ± 7.9 *(column 5)*, 11.3 ± 2.4 *(column 7)*, and 14.8 ± 5.7 *(column 9)*, respectively. While NS398 treatment by itself induced no *redBA/gam *loci mutations, its presence in the culture medium during either TiO_2 _nanoparticle or C_60 _treatment reduced the mutant fractions by 2.2-fold, 2.8-fold, and 2-fold to 7 ± 3.1 *(column 6)*, 4.1 ± 0.6 *(column 8)*, and 7.5 ± 4.5 *(column 10)*, respectively, for the 10 μg/ml dose treatment. In contrast, NS398 treatment had minimal effect on the mutagenic potential of TiO_2 _-325 mesh such that there was no decrease in mutant yield in cells treated with both NS398 and TiO_2 _-325 mesh as compared to those treated with TiO_2 _-325 mesh alone.

## Discussion

During the last few years, research on toxicologically relevant properties of manufactured nanoparticles has increased at an exponential rate. Currently, most of the toxicological work on nanoparticles have been generated with a small set of nanoparticles, such as carbon black, C_60_, TiO_2_, iron oxides and amorphous silica, which have been manufactured by the chemical industry for some decades and are produced in bulk quantities each year [5,37]. There is evidence that a number of factors are likely to contribute to the toxicity of nanoparticles, including particle number and size, surface area and charges, and chemical composition [38]. Nevertheless, experimental conditions, type or dose of nanoparticles used, or the nature of the assays can also modulate the assessment outcome. It is, therefore, necessary to establish an efficient system to determine the genotoxic events induced by nanoparticles both *in vivo *and *in vitro*.

Genetic alterations, such as point mutations, chromosomal rearrangements, recombination, and insertions or deletions of genes, are thought to be one of the earliest cellular responses caused by physical and chemical carcinogens and may play an important role in the initiation and progression of carcinogenesis [39]. Previous studies from this laboratory have shown that the *gpt *delta transgenic mouse system provides a unique opportunity to assess the mutagenic potential of asbestos fibers [30]. The *gpt *mice carry tandem repeats of λ G10 DNA in the chromosome, which are retrievable as phage particles by an *in vitro *packaging reaction. The rescued phages are then used to quantify the mutation yield upon exposure to genotoxic agents. The Spi^- ^selection based on deletions extending into or through both the *redBA *and *gam *genes is an efficient mutation assay system for detecting small to kilo-base-sized deletions in different cells, organs, and tissues [24]. Since gene mutation, mitotic recombination, chromosome loss, and interstitial deletion largely contribute to the development of malignancy, the establishment of the *gpt *delta transgenic mouse mutation model may provide new insight on understanding nanoparticle-induced mutagenesis. Our present findings demonstrated that TiO_2 _at nano-scale increased the mutant yield at the *gam *and *redBA *loci in MEF cells, while TiO_2 _at micro-scale had little effect on the mutation induction. These data were consistent with several *in vivo *and *in vitro *findings that, upon transition from the micro-scale to nano-scale size range, diameter of inhaled or instilled particles are important factors influencing the toxicity response [19,40,41]. The BET surface area for TiO_2 _5 nm was increased by 3-fold from 38.2268 m^2^/g to 114.1261 m^2^/g as compared to TiO_2 _40 nm, however, there was no statistically significant difference among groups expsoed to either TiO_2 _5 nm or TiO_2 _40 nm at the same dose (Figure 2), which are in conflict with the notion that toxic response is generally considered to be higher in particles with large surface area than those with smaller area [42]. Although a surface area dependence and correlation have been observed in instillation studies [5], recent evidence from rats and mice showed that the surface area for TiO_2 _nanoparticles was not a significant factor in inflammatory response [12,43]. In addition, we showed here that C_60 _was cytotoxic and mutagenic in transgenic MEF cells, although the exact mechanisms are largely unknown.

Endocytosis is a conserved process in eukaryotes by which extracellular components are taken up into cells by invagination of the plasma membrane to form vesicles that enclose these materials [44]. There are several possible uptake pathways for internalizing nanoparticles, such as phagocytosis, macropinocytosis, clathrin-mediated endocytosis, caveolae-mediated endocytosis, and clathrin-caveolae-independent endocytosis *(5, 45)*. Several recent evidence has shown that certain nanoparticles, such as iron oxide and silica, as well as carbon nanotubes, are internalized in cells via the endocytic pathway [46,47]. After 24 h incubation, we observed that the cellular granularity of MEF cells exposed to TiO_2 _particles was increased in a dose-dependant manner. In contrast, C_60 _had no effect on the cellular granularity, which might be due to their low contrast and small diameters. Our results with the lipid raft-disrupting agent Nystatin, which binds to cholesterol in cell membranes and disrupts the formation and trafficking of caveolae, provided further support of the idea that the endocytotic process modulated the mutagenic response of nanoparticle treatment [34]. Given C_60 _is lipophilic, it is possible that C_60 _may interact with plasma membrane lipids and exert toxicity directly in the absence of cellular uptake [18]. It is also likely that C_60 _interact with cell membrane receptors to trigger or alter intracellular signal transduction pathways. Due to high energetic adhesive forces close to the surface, nanoparticles are easily agglomerated to form larger particles. Thus, whether single particles or agglomerates are important in the genotoxicity of nanoparticles has not been identified yet.

The mechanism of oxidative stress induced by nanoparticles is not well understood. There is evidence that free radicals can be induced at the surface of nanoparticles such as single-wall carbon nanotube (SWCNT), semiconductor quantum dots, TiO_2_, environmental particles (e.g. PM-10), asbestos, and a range of man-made fibers [14,48,49]. Among the most biologically active oxyradicals such as superoxide anions (O_2_^.^), hydrogen peroxide (H_2_O_2_), and hydroxyl radical (OH^·^), NO is relatively long lived and catalyzed by nitric oxide synthase (NOS) [50]. The few cell culture experiments on nanoparticles, such as metal oxides and quantum dots, have identified particles within or around the mitochondria [17,33]. Since mitochondria constitute a major locus for the intracellular formation and reactions with NO, it is likely that multiple radical species are involved in the genotoxic response of TiO_2 _nanoparticle and C_60 _exposure. NO reacts with O_2_^-. ^and can be rapidly converted into more reactive nitrogen compounds such as ONOO^- ^that can cause nitration of proteins, hydroxylation or nitration of DNA, and mutations [51] Nano-sized TiO_2 _exposure has been reported to increase the production of NO and oxidative DNA damage in human bronchial epithelial cells [14]. In the present study, TiO_2 _nanoparticle exposure dramatically increased the generation of ONOO^- ^in MEF cells. It should be noted that nano-TiO_2 _particles in the anatase crystal phase were reported to be superior catalysts and more cytotoxic as compared to the rutile particle type, which might be due to differences inherent in the crystal structures of the two phases, rather than differences in surface area *(11)*. There is evidence that the unique structure of C_60 _facilitates absorption of light and transfer of this energy to triplet oxygen, thereby forming the highly reactive singlet oxygen state, which may cause oxidative damage in exposed organisms [52]. Recent reports have showed that C_60 _induces cytotoxic effects via the induction of reactive oxygen species in mouse cells, human cells, and fish. However, it should be noted that some data indirectly suggest that oxyradical-mediated cytoxicity of C_60 _might not be an inherent property of pure C_60_, but rather a result of the residual presence of tetrahydrofuran (THF), the organic solvent used for C_60 _preparation, which remains intercalated into its lattice [53]. Here, C_60 _suspension prepared by long-term stirring in water. The oxidation of DHR 123 by ONOO^-^, as detected using confocal microscopy, provided direct evidence that C_60 _induced a dose-dependent increase of ONOO^- ^in single cells, which could be inhibited by the NOS inhibitor L-NMMA. Moreover, the mutation yields induced by either nano-sized TiO_2 _or C_60 _in MEF cells decreased by concurrent treatment with L-NMMA, indicating a key role of ONOO^- ^in the mechanisms of nano-sized TiO_2 _and C_60_-induced genotoxicity. It's woth notice that the redox events might be caused by the signaling events associated with the transporting of naoparticles into the cellular structure, rather than the chemical composition/surface area combiantion of the nanoparticles.

COX-2 is a member of the COX family, which plays important roles in modulating cellular inflammation, carcinogenesis and genomic instability [39]. Nitric oxide synthase, which is critical to the biosynthesis of ONOO^-^, has been shown to be involved in the regulation of COX-2 expression [36,54]. Since COX-2 is the initial and rate-limiting enzymatic step in the metabolism of arachidonic acid into a complex group of signaling lipid mediators, the particle-induced oxidative stress may lead to transmit external signals into the cell and activate COX-2 signal pathway. In the presence of NS-398, a specific inhibitor of COX-2 [55], the genotoxic effects of both nano-sized TiO_2 _and C_60 _was reduced dramatically in MEF cells, thereby establishing the functional link for the role of ONOO^- ^and COX-2 in mediating the genotoxic events of both nano-sized TiO_2 _and C_60_.

The toxicological data specific to nanoparticles remains insufficient currently [5,56]. However, the potential toxicity of nanoparticles has attracted attention because of their apparent similarities to asbestos and other carcinogenic fibres/particles. Our present studies provided direct evidence on the genotoxicity of two specific types of manufactured nanoparticles, TiO_2 _and C_60_, and highlight several key health risk assessment issues associated with manufactured nanomaterial, such as the paucity of information on nanoparticle toxicology and exposure assessments as well as the extent to which nanoparticle toxicity can be extrapolated from existing particle and fiber toxicology databases.

## Abbreviations

TiO_2_: Titanium dioxide; C_60_: Fullerene; *gpt*: Xanthine phosphoribosyltransferase (NP_414773: GenBank); MEF: Mouse primary embryo fibroblast; DHR 123: Dihydrorhodamine 123; ONOO^-^: Peroxynitrite anions; O_2_^-^: Superoxide anions; COX-2: Cyclooxygenase-2; L-NMMA: N^G^-methyl-L-arginine; Spi^-^: Sensitive to P2 interference.

## Competing interests

The authors declare that they have no competing interests.

## Authors' contributions

XA carried out the preparation and performance of all experiments and wrote the paper. CYF assisted the Spi- matation determination. NT established the *gpt* delta mouse mutation assay system. HTK conceived and supervised the work.
